# Atypical Oscillating Pupillary Reaction in a Brainstem-Dead Patient

**DOI:** 10.7759/cureus.102410

**Published:** 2026-01-27

**Authors:** Mohamed Foda Hendi, Zeyad Faoor Medhat Alrais, Fahimuddin Syed, Ghaya Z Alrais, Mohamed H Elkhouly

**Affiliations:** 1 Intensive Care Unit, Rashid Hospital, Dubai, ARE; 2 Critical Care Medicine, Mohammed Bin Rashid University of Medicine and Health Science, Dubai, ARE; 3 Neurological Surgery, Dubai Medical College, Dubai, ARE; 4 Intern, Gulf Medical University, Ajman, ARE

**Keywords:** brain death, brainstem reflexes, eeg (electroencephalogram), organ donation, pupillary light reflex

## Abstract

Dilated and fixed pupils are a hallmark of the diagnosis of brainstem death. We present a case of a brain-dead patient whose pupil displayed unilateral spontaneous repetitive pupillary dilatation and constriction (in an oscillating manner) independent of external stimuli, which didn’t respond to light. Central causes of this phenomenon were excluded by confirmation of brainstem death via ancillary test, leaving the peripheral cause as the most likely explanation. This rare and atypical phenomenon is not a sign of brain activity and can be explained that the pupillary constrictor muscle can become hypersensitive after brain death, and it can be spontaneously constricted or dilated in response to circulating neurotransmitters in the blood. Also, the cilio-spinal reflex may be caused in brain-dead patients due to terminal neural discharges from dying cells, which lead to sympathetic stimulation of the ciliary ganglion. This case shows that oscillating pupillary activity in the absence of external stimuli should not interfere with or delay the diagnosis of brainstem death to facilitate earlier organ donation.

An oscillating spontaneous pupillary activity in a brain-dead patient is a rare, atypical phenomenon caused by a peripheral reflex rather than a central reflex. This atypical phenomenon should not interfere with or delay the diagnosis of brainstem death, which allows for proper and timely medical care and organ donation.

## Introduction

Dilated and fixed pupils are a hallmark of the diagnosis of brainstem death [1]. We report a rare case of atypical pupillary oscillations in a patient who met the criteria of brain death that might delay brain death certification. Loss of brainstem reflexes must be proven to confirm brainstem death, and the pupillary response to light is one of the essential brainstem reflexes [1,2].

We noticed a unilateral spontaneous repetitive pupillary dilatation and constriction (in an oscillating manner) independent of external stimuli, which didn't respond to light. The delay in diagnosing brain death results in distress to the family. The decision of organ donation may be delayed, affecting the suitability of organs needed for transplantation. Organ donation not only saves lives but also provides possibilities to improve the life quality for individuals with end-stage organ failure, and currently, the demand for organ donation far exceeds the availability of organs supplied [3].

Recognizing this atypical sign is crucial to avoid delays in diagnosing brain death, which allows for proper and timely medical care and organ donation.

## Case presentation

**Figure 1:**
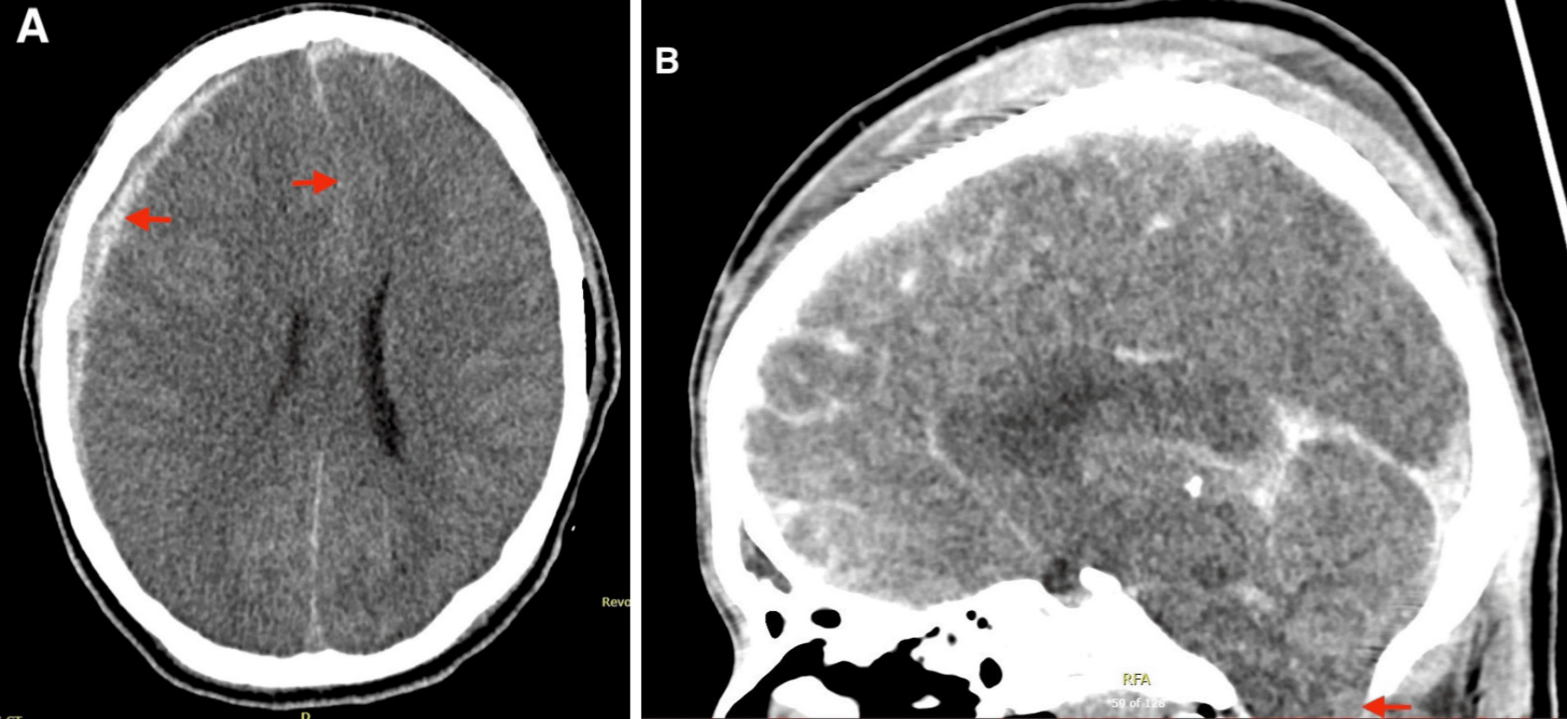
A) brain CT of the presenting patient showed a right-side subdural hematoma with midline shift; B) sagittal image demonstrates effacement of CSF space in the foramen magnum and tonsillar herniation CT - computed tomography; CSF - cerebrospinal fluid

A 17-year-old man arrived at the emergency department with multiple head injuries sustained from a road traffic accident when a scooter rider was hit by a frontal collision with a car. On arrival, the Glasgow Coma Scale was 3/15, pupils were fixed and dilated, and hemodynamically stable. He was intubated immediately in the emergency department and mechanically ventilated. He went for a computed tomography (CT) polytrauma scan that showed traumatic brain injury with a large right subdural hematoma associated with uncal herniation and a significant leftward midline shift (Figure 1), traumatic venous thrombosis of the left sigmoid and transverse sinus, subarachnoid hemorrhage of the right frontal and bilateral temporal space and a fracture at the base of the skull, extending into the left temporal bone. Other skeletal findings include dislocation of the left femoral head and bilateral fractures of the pubic symphysis.

The patient was referred to the neurosurgeon, who advised that he was not fit for surgical intervention and for ICU admission for medical management. On assessment, there was a cough reflex, but both pupils were dilated 5 mm and non-reactive bilaterally. 300 ml of mannitol was administered, and hyperventilation started.

A few hours later, both pupils were dilated and fixed with absent gag and cough reflexes. A follow-up CT brain showed an increase in the cerebral oedema with loss of grey-white matter differentiation. There was descending uncal as well as cerebellar tonsillar herniation with effacement of cerebrospinal fluid spaces. The neurosurgeon's decision was not for surgical intervention, as the neurological condition did not change after mannitol.

**Figure 2:**
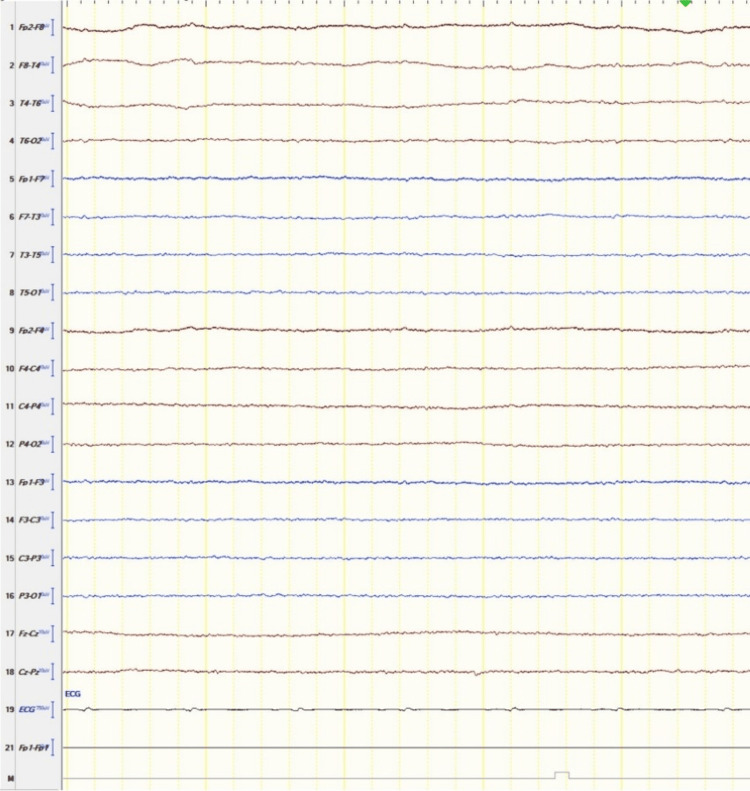
Electroencephalogram (EEG) of the presenting patient showed iso-electric cerebral activity (electrocerebral silence)

After excluding potential reversible causes for coma, the first test of brainstem reflexes was performed approximately 24 hours after admission, and all the reflexes were absent. The patient was retested by a neurologist after a 12-hour interval. On re-testing, all the brainstem reflexes were absent, and the pupils were unequal and non-reactive to light. An electroencephalogram (EEG) showed profound generalized suppression, indicating severe diffuse nonspecific neocortical cerebral dysfunction (a pattern of electrocerebral silence) (Figure 2).

The next day, we noticed a change in the right pupil size; it was found to be mid-dilated (4 mm) and active, but not in response to light, while the left pupil was larger (6 mm). The right pupil showed continuous repetitive pupillary dilatation and constriction, which was unrelated to light, while the other pupil was dilated and fixed. This rare phenomenon was continuously observed over the next 24 hours and recorded on video (Video 1, Video 2). This unilateral spontaneous pupillary activity was misinterpreted initially as a reaction to the light. Brain death declaration was postponed until one further test of absent brainstem reflexes was confirmed, and an EEG was repeated, showing no cerebral activity. The patient was declared brain-dead more than 36 hours after the initial brainstem death tests. This rare phenomenon of continuous repetitive dilatation and constriction of the right pupil was noticed frequently for the next days before the patient's organs were used for transplantation.

**Video 1:** A video recorded of repetitive constriction and dilatation of the right pupil under constant illumination

**Video 2:** Another video recorded of repetitive constriction and dilatation of the right pupil of presenting patient under constant illumination

## Discussion

Organ donation not only saves lives but also provides possibilities to improve the life quality for individuals with end-stage organ failure. Currently, the demand for organ donation far exceeds the availability of organs supplied.

Human Organs & Tissues Donation Services support saving lives through organ donation by identifying potential donor candidates using brain death by neurological criteria (DNS) [1]. DNS is defined as the irreversible loss of consciousness along with the irreversible termination of all brain and brainstem activities. Brain death judged by neurological criteria is comparable to the person's death, even if spinal cord functions persist and the heart continues to beat [1].

DNS is based on a set of criteria for assessing and diagnosing brainstem death [2]. Strict criteria were placed for the brain death diagnosis, which could be made at the bedside, without the need for special investigations (ancillary tests such as transcranial Doppler or CT cerebral angiogram). The three essential characteristics of brainstem death are coma, loss of brainstem reflexes, and apnoea [4].

Certain preconditions must be met before the assessment of brainstem reflexes. The patient is in a deep coma for a known reason, and the event that caused the coma occurred at least six hours prior. To rule out reversible causes of coma, such as hypothermia, drug intoxication, and metabolic and endocrine disturbances, biochemical tests should be performed, and the patient should not be under the effect of any anxiolytics, sedatives, narcotics, hypnotics, muscle relaxants, or anti-depressants [2].

Other preconditions before brainstem reflexes assessment include that the patient is not in untreated cardiovascular shock, has a core temperature above 36°C, can't initiate spontaneous ventilation, and is dependent on mechanical ventilation [4].

Loss of brainstem reflexes, including the pupillary response to light, must be proven [1]. The patient should not respond to any kind of stimuli, with the possibility of having some minimal spinal cord reflexes. It is recommended that the brainstem reflexes test be performed twice, typically a few hours apart. If the two tests have been completed without constraints, the apnoea test should be performed to verify the absence of brainstem reflexes and confirm brain death. If no respiratory effort is seen with a PaCO₂ >60 mmHg, it can be assumed the patient is brainstem-dead [3].

The ancillary test is not necessary; it is employed only if the clinical exam parts or apnoea test can't be completed. If the two clinical examinations or the apnoea test can't be completed for any reason, then it is required to perform one of the ancillary tests, e.g., an EEG, transcranial Doppler, or CT cerebral angiogram [4,5].

The pupillary reflex to light is one of the essential brainstem reflexes [2]. The pupillary reaction to light needs the presence of an intact reflex arc passing through the brainstem. The afferent component fibers pass to the nucleus in the midbrain via the antero-visual pathway. The efferent fibers pass via the oculomotor nerve, which synapses in the ciliary ganglion in the orbit that supplies the pupillary sphincter. Total loss of responsiveness to light can result from complete disruption of either afferent and/or efferent pathways, as well as brainstem damage [6].

This patient had irreversible brain injury, which was confirmed by a CT scan, while all possible reversible causes of coma had been excluded. He met the preconditions and criteria for brainstem death. Despite the absence of brainstem reflexes and respiratory function, unilateral spontaneous pupillary activity was still demonstrated.

In this patient, spontaneous pupillary activity of the right pupil was initially misinterpreted as a response to light. It was noticed that there were repetitive pupillary constrictions and dilatations. The right pupil dilates and contracts rhythmically and spontaneously, independent of head movements. After a period of uninterrupted observation, it was displayed that the pupils were unresponsive to light.

This unilateral oscillating pupillary activity is unlikely to be a central response, as all the sympathetic and parasympathetic activity is lost in brainstem death. Central reflex is typically synchronous and bilateral (bilateral, simultaneous fluctuation in pupil size) [6]. Furthermore, for a centrally originated stimulus to elicit a pupillary response to light, an intact efferent pathway is required, which was not present in this patient due to brain herniation, which compressed the oculomotor nerve against the tentorium edge, as shown radiologically (CT).

Central causes of this phenomenon were excluded by confirmation of brain death via an ancillary test (EEG). After brain death, the brain's control over the pupils is lost. This rare and atypical phenomenon is not a sign of brain activity, leaving a peripheral cause as the most likely explanation.

The unilateral oscillating pupillary activity must originate peripherally, arising either in the pupillary sphincter or the ciliary ganglion, and can be explained by the fact that the pupillary constrictor muscles can become hypersensitive after brain death and can be spontaneously constricted or dilated in response to circulating neurotransmitters in the blood [7]. Another explanation is that both pupillary dilatation and constriction may occur due to a cilio-spinal reflex in patients with brain death [8]. Cilio-spinal reflex may be caused in brain-dead patients due to terminal neural discharges from dying cells, which lead to sympathetic stimulation of the ciliary ganglion [7]. We can't fully explain this rare phenomenon, but spontaneous pupillary activity produced by intermittent discharges of dying neurons is similar to Cheyne-Stokes respiration seen in brainstem damage [9].

Pupillary reflexes to light are typically bilateral and fixed, with pupils larger than 4 mm in diameter. Changes in pupil diameter in response to the stimulation of peripheral nerves are sometimes observed [10]. Only two studies had reported abnormal unilateral pupillary movement in brain-dead patients [6,10]. This atypical phenomenon requires additional research to determine its pathophysiology [11].

This spontaneous pupillary activity initially was interpreted as a response to light. It emphasizes the importance of the pupillary reflex technique. It is important to have a period of uninterrupted observation during pupil testing.

## Conclusions

This case shows that oscillating pupillary activity in the absence of external stimuli should not interfere with and delay the diagnosis of brain death. Recognizing this atypical sign is crucial to avoid delays in diagnosing brain death, which allows for proper and timely medical care and can facilitate earlier organ donation.
